# The Clinical Significance and Translational Implications of Subclinical Interstitial Lung Abnormalities in Asymptomatic Adults: A Narrative Review

**DOI:** 10.7759/cureus.92255

**Published:** 2025-09-14

**Authors:** Julian Eduardo Bedoya Jaramillo, Richard Adrian Vergara Trujillo, Jenny Tatiana Alarcón Plaza, Angie Carolin Vanegas Wilches, Bryan Nicolás Forero Vásquez, Walfred Ramiro Osorio Reyes

**Affiliations:** 1 Internal Medicine, Universidad San Sebastián, Puerto Montt, CHL; 2 Nephrology, DaVita Colombia, Cali, COL; 3 General Practice, ESE Hospital San Francisco, Villa de Leyva, COL; 4 Critical Care Medicine, Sociedad de Cirugía de Bogotá - Hospital de San José, Bogotá, COL; 5 Internal Medicine, Fundación Hospital San Carlos, Bogotá, COL; 6 Internal Medicine, Hospital Nacional de Jutiapa, Jutiapa, GTM

**Keywords:** asymptomatic adults, clinical significance, narrative review, subclinical interstitial lung abnormalities, translational implications

## Abstract

This literature reports that there is a difference between interstitial lung abnormalities (ILAs) and subclinical interstitial lung abnormalities (ILAs). The objective of this narrative review was to comprehensively summarize the current knowledge on the ILA spectrum, particularly subclinical ILA. We aimed to clarify the nuanced definitions and prevalence of ILAs in asymptomatic adults, delineate their recognized risk factors, and explore the histopathological and genetic underpinnings of these conditions. This narrative literature review was conducted using relevant topic-specific keywords such as “Interstitial Lung Disease,” “Subclinical Interstitial Lung Disease,” “Subclinical Interstitial Lung Abnormalities,” “Interstitial Lung Abnormalities,” “Progression of Interstitial Lung Disease,” “Subclinical Interstitial Lung Disease (ILD),” “Subclinical ILA,” “Asymptomatic adults,” “Computational tomography,” “Incidental CT scan,” “Serial CHEST CT,” “Disease Progression,” “Risk Factors,” “Mortality,” “Lung Function Tests,” and “Genetic Factors.” Boolean operators were used to incorporate the keywords and search on PubMed and Google Scholar, focusing on full-text open-access articles from January 2015 to July 2025. In asymptomatic adults, subclinical interstitial lung abnormalities (ILAs) were considered incidental findings. It is now increasingly being recognized that these are at least predisposing factors to early-stage interstitial lung disease, yet their clinical implications are unclear. The incidence rises with age and exposure to smoking; however, much of that evidence lacks longitudinal follow-up studies that are detailed enough to distinguish mild ILAs with benign courses and progressive disease-producing ILAs. The asymptomatic ILA management is not standardized because there is a lack of universally accepted diagnostic criteria, risk stratification assessment tools, and clearly formulated instructions referring to surveillance. To transform ILAs as incident discoveries into some clinically actionable data, there is a need to widen the views by prospective studies, the generation of biomarkers, and investigation in long-term follow-ups.

## Introduction and background

Interstitial lung abnormalities (ILAs) are findings on high-resolution computed tomography (HRCT) that suggest underlying interstitial lung disease (ILD) or early-stage pulmonary fibrosis [1]. They are defined as non-dependent abnormalities affecting more than 5% of any lung zone, including features like ground-glass or reticular abnormalities, non-emphysematous cysts, honeycombing, or traction bronchiectasis [2]. The increasing use and improving quality of CT imaging, particularly through lung cancer screening programs and health screening cohorts, have significantly increased the incidental detection of ILAs [3,4].

ILAs and subclinical ILAs differ, according to current literature. Accidental chest CT imaging abnormalities indicate interstitial lung disease (ILD) or early pulmonary fibrosis (PF) in persons without a clinical diagnosis. Studies link ILA to poor lung function, exercise ability, and survival, suggesting some people with ILA may have an unintentional but clinically important ILD [5]. Few have risk-stratified ILA utilizing CT characteristics and lung function indicators to identify a subpopulation with suspected or subclinical ILA [6,7]. Instead, these accidentally identified pulmonary changes in asymptomatic individuals with preserved lung function are called subclinical ILA [8].

ILA is usually detected in cohorts, which may include patients with limited pulmonary function impairment who were accidentally identified on imaging while being asymptomatic. These clinically significant findings have led to the reclassification of ILA as “mild ILD” rather than subclinical ILA. This suggests that ILA, early ILD, and mild ILD may form a continuum of ILD progression [9].

Why asymptomatic adults matter? Despite often being asymptomatic at the time of detection, the recognition of ILAs in otherwise healthy adults is gaining increasing clinical importance. This is because a significant subset of individuals with ILAs, particularly those with fibrotic patterns, are at increased risk of progression towards clinically overt ILD [10]. Therefore, understanding ILAs in asymptomatic adults is crucial for identifying those at greatest risk and potentially intervening before the development of more severe, symptomatic disease.

Why subclinical ILAs may offer a preclinical window into ILD? Growing evidence suggests that subclinical or mild or pre-clinical ILAs may indeed represent an early stage of ILD [11]. There are striking similarities between ILAs and clinically overt ILD, including overlapping genetic risk factors (e.g., the MUC5B promoter polymorphism, a common and significant genetic risk locus for idiopathic pulmonary fibrosis (IPF), similar physiological decrements (though often less severe), and shared histopathologic findings [12]. The progression of ILAs is also associated with an accelerated decline in pulmonary function and an increased risk of death, mirroring the trajectory of established ILD, like IPF. ILAs can identify a preclinical window for early intervention and risk factor modification in IPF, a relentlessly progressing and fatal illness that is actively treated even in its early stages [13].

The objective of this narrative review was to comprehensively summarize the current knowledge on ILA's spectrum, particularly subclinical ILA. We aimed to clarify the nuanced definitions and prevalence of ILAs in asymptomatic adults, delineate their recognized risk factors, and explore the histopathological and genetic underpinnings of these conditions. Crucially, this review will highlight the need to define those individuals at greatest risk of progression to clinically significant ILD and adverse clinical outcomes, including lung cancer and mortality. By narratively synthesizing existing data, we seek to underscore the importance of early recognition, appropriate monitoring strategies, and the potential for timely interventions to modify disease course and improve patient outcomes.

## Review

Methodology

This narrative literature review was conducted using relevant topic-specific keywords such as “Interstitial Lung Disease,” “Subclinical Interstitial Lung Disease,” “Subclinical Interstitial Lung Abnormalities,” “Interstitial Lung Abnormalities,” “Progression of Interstitial Lung Disease,” “Subclinical ILD,” “Subclinical ILA,” “Asymptomatic adults,” “Computational tomography,” “Incidental CT scan,” “Serial CHEST CT,” “Disease Progression,” “Risk Factors,” “Mortality,” “Lung Function Tests,” and “Genetic Factors.” Boolean operators were used to incorporate the keywords and search on PubMed and Google Scholar, focusing on full-text open-access articles from January 2015 to July 2025. The literature search was limited to peer-reviewed literature in the English language. Data extraction included characteristics from the included studies, such as author, year, study design, cohorts, definition and classification of ILA, prevalence, incidence, progression of ILD, risk factors, mortality, and screening.

Narrative synthesis

Definition and Classification of ILAs

ILAs are non-dependent CT anomalies that affect more than 5% of any lung zone (upper, middle, or lower lung zones, delimited by the inferior aortic arch and right inferior pulmonary vein). Ground-glass opacities, reticular or nodular abnormalities, lung deformation, honeycombing, traction bronchiectasis, and non-emphysematous cysts are characteristic abnormalities of ILA [1,2,14]. ILAs are different from dependent lung atelectasis, focal paraspinal fibrosis next to osteophytes, mild focal or unilateral abnormalities, interstitial edema (e.g., heart failure), pleuroparenchymal fibroelastosis elements, aspiration, or smoking-related centrilobular nodularity without other interstitial characteristics [15].

Fleischner Society Position Paper (2020) on ILA Subtypes

**Figure 1:**
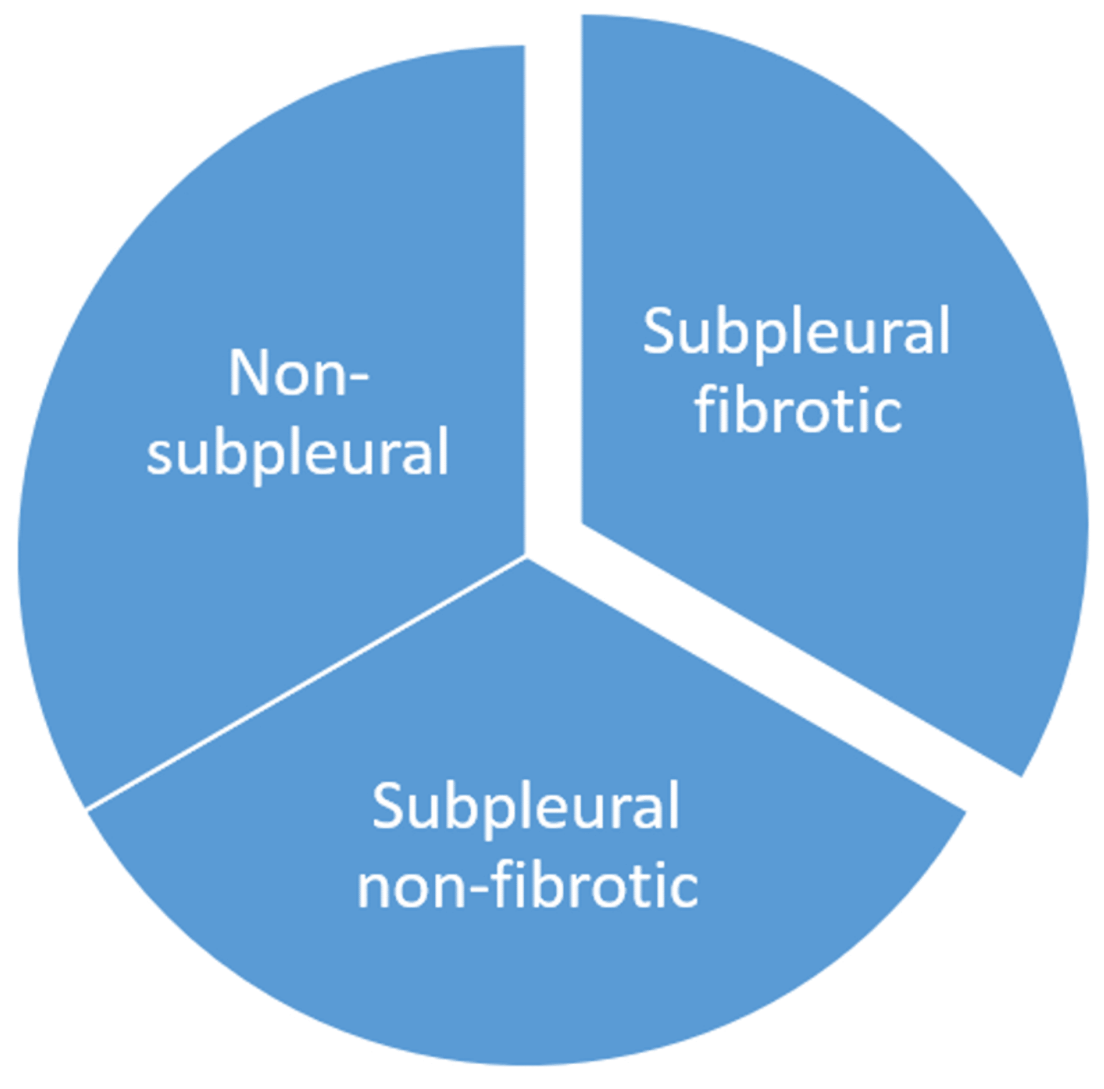
Interstitial lung abnormalities (ILAs) subtypes. This image is created by the authors of this study.

The Fleischner Society standardized ILA terminology by prognostic relevance. Figure 1 shows non-subpleural, non-fibrotic, and fibrotic ILAs. Non-subpleural ILAs often present with ground-glass and reticular opacities. These rarely progress and are generally not fatal. Subpleural non-fibrotic ILAs have ground-glass and reticular opacities with considerable subpleural localization but no fibrosis. Reticulation in this subtype increases the risk of progression. Subpleural fibrotic ILAs feature traction bronchiectasis, architectural deformation, and honeycombing and are subpleural. They greatly accelerate ILA development and mortality [7].

Clarification of Subclinical ILAs Within the ILA Spectrum

ILA is a radiological term for accidental identification in people without ILD. ILA does not equal subclinical ILD. Asymptomatic patients with preserved lung function are diagnosed and classified as subclinical, preclinical, or early ILD according to current criteria [5-7]. A 70-year-old man with asymptomatic reticular subpleural abnormalities and traction bronchiectasis (ILA) was reclassified as early idiopathic pulmonary fibrosis (IPF) after lung biopsy and progressed over several years. This suggests that ILA can develop and evolve with ILD [16].

Imaging-Based Detection Thresholds

The usual definition of ILA involves visual abnormalities in more than 5% of any lung zone; however, this threshold may be arbitrary [17]. Quantitative methods using deep learning-based texture analysis have suggested lower optimal cutoffs for ILA detection, for instance, 1.8% of the lung area in a Korean lung cancer screening cohort, showing 100% sensitivity and 99% specificity. This quantitative approach offers objective identification and may help reduce interobserver variability. Inter-reader agreement for ILA status can be excellent (kappa=0.93) and good for ILA subtype determination (kappa=0.76). Single-observer evaluations have also shown good intra- and inter-reader agreement (92% and 83.5%, respectively) [18]. A comparison of the radiologic definition of ILA used by major cohorts is presented in Table 1.

**Table 1:** Comparison of radiologic definitions used in major cohort studies. AGES: age, gene/environment susceptibility; COPD: chronic obstructive pulmonary disease; ECLIPSE: Evaluation of COPD Longitudinally to Identify Predictive Surrogate End-points; FHS: Framingham Heart Study; ILD: interstitial lung disease; MESA: multi-ethnic study of atherosclerosis; HU: Hounsfield units; HAA: high attenuation areas; ILA: interstitial lung abnormalities

Study/cohort	ILA definition	Key nuances/thresholds
Framingham Heart Study (FHS) [19,20]	Non-dependent changes affecting more than 5% of any lung zone. Includes ground-glass or reticular abnormalities, diffuse centrilobular nodularity, non-emphysematous cysts, honeycombing, or traction bronchiectasis.	Focal or unilateral ground-glass attenuation, reticulation, or patchy abnormalities (<5% of lung) were considered indeterminate, not ILA.
Multi-Ethnic Study of Atherosclerosis (MESA) [17,21]	Visual ILA: same as FHS: non-dependent changes >5% of non-dependent lung by reticular, ground-glass, diffuse centrilobular nodularity, honeycombing, traction bronchiectasis, and/or non-emphysematous cysts. Quantitative HAA: percent of imaged lung volume with CT attenuation between -600 and -250 HU.	Indeterminate if patchy, focal, or unilateral abnormalities. HAA is a quantitative measure correlated with ILD.
COPD Gene Study [22,23]	Non-dependent changes involving over 5% of any lobar region. Ground-glass or reticular abnormalities, diffuse centrilobular nodularity, non-emphysematous cysts, honeycombing, or traction bronchiectasis.	Excludes those with centrilobular nodularity alone. Indeterminate if focal or unilateral ground-glass attenuation or reticulation, and patchy ground-glass abnormalities.
ECLIPSE Study [23]	Evaluated by automated CT methodologies. The general ILA definition aligns with the Fleischner Society consensus.	Included in large cohorts evaluated by automated methods.
AGES-Reykjavik Study [24]	Non-dependent changes affecting more than 5% of any lung zone. Includes ground-glass or reticular abnormalities, diffuse centrilobular nodularity, non-emphysematous cysts, honeycombing, and traction bronchiectasis.	Changes in <5% of lung zones or focal/unilateral/patchy abnormalities were indeterminate.
Korean National Lung Cancer Screening Program [18]	Non-dependent abnormalities affecting at least 5% of any lung zone, including ground-glass or reticular abnormalities, non-emphysematous cysts, traction bronchiectasis, or honeycombing.	Equivocal ILA is defined as affecting less than 5% or unilateral abnormality. Deep learning suggests an optimal cutoff of 1.8% for quantitative detection.

Epidemiology and prevalence

The prevalence of ILAs varies depending on the population studied, but they are relatively common, with estimates ranging from 2% to 10% in the general adult population [8,25]. In specific cohorts, such as the Framingham Heart Study (FHS), ILAs were seen in 7% of participants. Their prevalence increases with age, being higher in older adults (e.g., up to 17% in patients with a mean age of 78 years and up to 47% in those aged 70 years or older in the FHS). They are also more prevalent in smokers, with reported rates of 4% to 9% in cigarette smokers, and higher with increasing tobacco exposure. Even in never-smokers, ILAs are common, especially the non-fibrotic phenotype [9]. In smokers and lung cancer screening cohorts, ILA prevalence is also elevated, ranging from 4% to 9% in smokers and 4% to 20% in screening cohorts [26,27]. Moreover, the COPD Gene study found ILA in 10% of its phase 2 participants. This increased prevalence is observed even in younger smoking cohorts (mean age 60-66 years) compared to general population cohorts [22].

Emerging Prevalence Data From Routine Health Check-Up CTs in Asia

Studies from Asian populations, particularly in Korea and Japan, offer valuable insights into the prevalence of ILA in mass screening programs. In the Korean National Lung Cancer Screening Program, the prevalence of ILA was approximately 4% among participants, predominantly heavy smokers. This figure is slightly lower than some reports from Western countries [18]. An Asian health screening cohort study reported an ILA prevalence of about 3% among individuals aged 50 years or older, with a mean age of 59 years. Notably, 80% of patients with ILA in this cohort showed progression over a median CT follow-up of eight years. This highlights the clinical relevance of these incidental findings even in younger Asian cohorts [4].

Risk factors and genetic associations

Environmental Exposures and Host Factors

Smoking is the strongest risk factor for ILA, rising with tobacco exposure or active smoking. The prevalence of spirometry restriction and high-attenuation areas (HAA) in the lung increases with higher pack-years of cigarette smoking [9,28]. Exposure to ambient air pollution, including elemental carbon (EC), a traffic-related fine particulate matter (PM2.5) constituent, is associated with increased odds of ILA. A five-year EC exposure (IQR difference of 0.14 µg/m³) was linked to 1.27 times greater odds of ILA and 1.33 times greater odds of ILA progression. Nitrogen oxides (NOx) also showed associations with ILA risk, especially in non-smokers [29]. Self-reported occupational vapor, gas, dust, and fume exposures are linked to preclinical ILD or ILA. Current workers and those under 65 years of age with such exposures have a higher risk of ILA. Low asbestos exposure can cause individuals with ILAs to meet the criteria for asbestosis [21]. ILA occurs more often with age; with each decade of age, ILA risk increases by 2.2-fold. In smokers with ILA, male gender increases prevalence 1.7-fold [9,28].

Comorbidities

ILAs are associated with paraseptal emphysema. While some studies show no direct link with COPD, others indicate that COPD patients with ILAs have a higher rate of exacerbations and accelerated lung function decline. Elements of autoimmunity (e.g., positive antinuclear antibodies) have been found in COPD patients with ILA [22,23,30]. Moreover, ILAs are a significant risk factor for lung cancer incidence and mortality. Fibrotic ILA specifically confers a higher risk. ILAs are also associated with increased postoperative pulmonary complications, radiation pneumonitis, and immune checkpoint inhibitor-induced pulmonary toxicity in cancer patients [31]. Subclinical lung abnormalities are frequent in anti-citrullinated peptide antibodies (ACPA)-positive RA patients, even before arthritis onset. Older age, male sex, and smoking are associated with ILA/ILD in RA. Patients with RA and ILA often present with respiratory symptoms, restrictive pulmonary function patterns, and reduced exercise capacity. Elevated serum levels of surfactant protein D (SPD) are associated with subclinical lung involvement and HRCT abnormalities in RA [32]. Lastly, ILA has been associated with obstructive sleep apnea [33].

Genetic Variants

MUC5B promoter polymorphism (rs35705950): This is the most common and highest genetic risk locus for IPF, and it is strongly associated with ILA. This variation raised the risks of ILA by 2.8 times and definitive CT evidence of pulmonary fibrosis by 6.3 times in the Framingham Heart Study. It is connected to the serial progression of ILAs, particularly the subpleural subtype and a typical interstitial pneumonia (UIP) pattern [24].

Other genes: Common genetic variants at loci such as DPP9, DSP, and FAM13A have shown significant associations with both ILA and IPF. Additionally, findings from in vitro diagnostic (IVD) studies have supported these genetic links [34]. Additionally, novel genetic associations for ILA, unrelated to IPF, have been discovered near IPO11 and FCF1P3 for general ILA and near HTRE1 for subpleural-predominant ILAs [9]. These findings suggest both shared and distinct genetically driven biological pathways between ILA and IPF.

Polygenic Risk Scoring and Machine Learning Risk Stratification Models for ILA Prediction

Polygenic risk scores (PRS): While the MUC5B variant is a significant individual predictor, polygenic risk scores that include other genetic variants (even without the MUC5B region) have also been developed for IPF. MUC5B and polygenic risk scores are associated with ILA and ILA progression. These ratings can identify high-risk patients for interstitial lung abnormalities and pulmonary fibrosis [9,12].

Machine learning and deep learning: These methods are increasingly applied for the automatic identification and classification of ILA patterns from CT images. A deep learning-based texture analysis in a Korean cohort showed 100% sensitivity and 99% specificity for detecting ILA using a 1.8% lung area cutoff value. This technology provides a more objective and consistent recognition of ILAs, which can help reduce interobserver variability in radiology reports. Deep learning-based ILA quantification showed that the median time to radiologic progression of ILA was 3.2 years and that the percentage of fibrotic ILA in the entire lung was an independent risk factor for ILA progression and UIP development [9,18].

Radiologic and pathologic features

CT Appearance of Subclinical ILAs

Subclinical ILAs on CT have ground-glass opacities, reticulations, and bronchiectasis, indicating fibrosis and architectural distortion. Honeycombing, widespread centrilobular nodularity, and non-emphysematous cysts are further hallmarks. These findings are sometimes detected accidentally during scanning of patients [1,2,13].

Fibrotic vs. Non-fibrotic ILA Distinction

Due to prognostic implications, fibrotic and non-fibrotic ILAs must be distinguished. Architectural deformation, tension bronchiectasis, or honeycombing indicate pulmonary fibrosis in fibrotic ILA. These patterns - particularly subpleural reticular marks, traction bronchiectasis, and honeycombing - increase ILA progression and mortality. Honeycombing progresses over five years in all situations. ILA without fibrosis has ground-glass and reticular opacities. The majority of non-subpleural ILAs are non-progressive and do not jeopardize survival [4,9,12].

Pathologic Overlap with Early IPF

ILAs are considered by some to be an early stage of ILD, including IPF. Histopathological studies of lung tissue specimens, often obtained during lung cancer resections, reveal that ILAs share overlapping features with IPF. The majority of patients with ILA in these specimens exhibited smoking-related interstitial fibrosis. Other interstitial patterns include typical interstitial pneumonia (UIP), pulmonary Langerhans cell histiocytosis (PLCH), and non-specific interstitial pneumonia (NSIP). UIP pattern was histologically found in 7-8% of ILA cases. ILA is significantly linked to subpleural fibrosis, fibroblastic foci, and atypical adenomatous hyperplasia histologically. In some situations, CT scans may not capture histological fibrosis, underestimating pathological ILD. Histopathology studies of subpleural fibrotic and progressive ILA are urgently needed to explain these relationships [10,12,27,28].

Progression From Subclinical ILA to IPF

The progression of lung abnormalities often follows a continuum, starting from incidental findings and potentially developing into advanced disease. This progression highlights the importance of early detection and management [19]. Interstitial lung abnormalities (ILA) are the initial stage, characterized by incidental CT abnormalities affecting more than 5% of any lung zone, often without noticeable symptoms or significant pulmonary function impairment. For instance, a patient might present with reticular subpleural abnormalities and mild traction bronchiectasis, yet have normal forced vital capacity (FVC) and diffusing capacity of the lung for carbon monoxide (DLCO), and be asymptomatic [1,2,13].

In some cases, ILA, particularly fibrotic subpleural ILA, can represent subclinical idiopathic pulmonary fibrosis (IPF). This stage is characterized by ascertained ILD (e.g., a definite usual interstitial pneumonia pattern on histology) in asymptomatic patients with preserved lung function. At this stage, while lung function might still be near normal (e.g., FVC ~120%, DLCO ~73%), a lung biopsy might show a definite usual interstitial pneumonia (UIP) pattern, leading to reclassification [4,9,12,19]. As the disease progresses, patients may experience mild symptoms or trivial pulmonary function impairment. Although symptoms are mild, lung function tests may show some decline (e.g., DLCO dropping to 73%). This stage is still considered clinically significant ILD [4,9,12,19].

**Figure 2:**
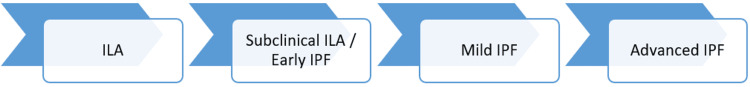
Progression of interstitial lung disease (ILD). IPF: idiopathic pulmonary fibrosis; ILA: interstitial lung abnormalities This image is created by the authors of this study.

Advanced IPF represents the later stage of the disease, where patients become symptomatic with significant declines in pulmonary function (e.g., FVC dropping to 60%, DLCO to 40%). Even early-stage IPF is aggressively treated because it is progressive and fatal. This progression from incidental radiological findings to overt clinical disease underscores the potential for early intervention if high-risk ILA subgroups can be accurately identified. Interstitial lung abnormalities (ILAs) are increasingly recognized as an important entity with significant implications for patient prognosis and clinical management. Figure 2 illustrates the progression of ILD [4,9,12,19].

Natural history and progression

Data from large population cohorts shed light on the progression rates of ILAs. The Framingham Heart Study (FHS) included a cohort of 1,867 participants with serial CT scans over approximately six years; 6% had ILA progression or developed new ILAs. Specifically, 43% of participants with ILA on their initial CT scan showed progression over the approximately six-year period. Progression in the FHS was associated with an accelerated decline in pulmonary function and an increased risk of death [13]. In the Multi-Ethnic Study of Atherosclerosis (MESA)*, *the incidence of fibrotic ILA was 3.5 cases per 1,000 person-years in a multi-ethnic population over 45 years of age. The overall incidence of new ILA cases in MESA was 13.1 per 1,000 person-years [17]. In the COPDGene Suspected ILD Cohort, approximately 54% of participants with ILA were classified as having "suspected ILD" based on advanced radiological findings and/or reduced pulmonary function. This suspected ILD group had higher mortality, reduced functional status, increased symptoms, greater oxygen use, severe respiratory exacerbations, and lower functional status. Compared to 6% of ILA patients without suspected ILD, 15% of those with probable ILD died [22].

General Progression Rates

The rate of CT progression for ILAs is estimated at 20% over two years and 48% over five years [9]. A systematic review and meta-analysis reported an overall pooled progression rate of 47.1%, with rates significantly higher in longer follow-up studies (31.0% in <4.5 years vs. 64.2% in ≥4.5 years) [12]. Lack of longitudinal follow-up was observed in subpleural non-fibrotic ILA-related studies. While fibrotic ILA patterns are extensively studied and clearly linked to progression and worse outcomes, the natural history of subpleural non-fibrotic ILAs is less clear. These were the most frequent ILA subgroup in one report (48% of cases) [35]. Although reticulation is noted as a major risk factor for progression in subpleural non-fibrotic ILA, with almost half progressing in four years in a Chinese cohort [36], the overall understanding of their long-term trajectory and the precise proportion that progress to clinically significant ILD remains to be clarified through dedicated longitudinal studies [9,12].

Clinical significance

ILAs are not benign findings and carry significant clinical implications, even in asymptomatic adults. ILAs are associated with accelerated declines in pulmonary function, including forced vital capacity (FVC) and diffusing capacity of the lung for carbon monoxide (DLCO) [9,12]. For instance, progressive ILAs in the FHS were associated with an excess FVC decline of 30-35 mL per year compared to those without ILAs. Patients with ILA progression lost 64 mL/year of FVC. DLCO reduction is also a significant physiological decrement observed in ILA [37].

ILAs significantly increase all-cause mortality. Progressive ILA was predictive of a fourfold increase in the risk of death in the FHS [20]. In an Asian cohort, fibrotic ILA had a 36% mortality rate within 10 years and was independently associated with an all-cause mortality hazard ratio (HR) of 2.5 and a disease-specific mortality HR of 6.7. The increased mortality risk among those with ILAs is too great to be solely explained by undiagnosed progressive ILD, suggesting other contributing factors [18].

ILAs are linked to lung cancer, cancer mortality, acute exacerbations, respiratory failure, sleep-related breathing difficulties, COPD, emphysema, and pneumonia. ILAs also increase the risk of postoperative pulmonary complications after lung cancer surgery [30]. Moreover, ILAs are linked with accelerated aging and associated plasma biomarkers like growth differentiation factor 15 (GF-15), tumor necrosis factor α receptor II (TNFR), interleukin-6 (IL-6), and C-reactive protein (CRP) [38]. Increased plasma GF-15 levels highly predicted ILA presence and mortality [39]. This shows that ILA and aging share pathogenic mechanisms. Recent discoveries suggest ILAs may be linked to heart failure and non-lung malignancies.

Introducing the Concept of “Silent Lung Injury” and Its Systemic Consequences

ILAs often represent a "silent lung injury" because they are incidental CT findings in individuals who may be asymptomatic or have mild, overlooked symptoms [9]. Despite this subclinical presentation, the presence of ILAs indicates ongoing pathological processes that can lead to systemic consequences [12]. This includes chronic inflammation, as evidenced by elevated pro-inflammatory biomarkers like resistin, MMPs, IL-6, and CRP in ILA patients. Furthermore, ILAs are strongly linked to accelerated aging, with specific plasma biomarkers of aging like GF-15 showing strong associations with ILA and mortality [38]. This connection suggests that ILAs are not merely isolated lung findings but reflect broader systemic changes that contribute to overall health decline and increased mortality, even in the absence of overt respiratory symptoms. Quantitative CT measures of pulmonary vascular pruning (loss of small vessels) are also associated with ILAs and ILA progression, suggesting early pulmonary vasculopathy that may be detectable before traditional physiological criteria for interstitial disease are met [9,12,17,18,22,38]. This highlights that ILAs can have a subtle yet significant systemic impact on the body, extending beyond just the lungs.

Implications for screening and clinical practice

The growing recognition of ILA's clinical significance raises critical questions for screening and clinical practice. The question is, should subclinical ILAs be monitored? The sources strongly recommend monitoring ILAs, especially fibrotic subtypes and those at risk of progression. An expert panel agreed that early ILA detection and follow-up will lead to improved outcomes. Since ILA affects 7% of the population, monitoring those cases at risk of advancement seems acceptable. Nevertheless, pulmonologists should diagnose and treat ILD early when necessary [9,10].

Another question is, what is the role of the following tests and imaging? The answer is that serial CT scans are essential for ILA progression evaluation. The Fleischner Society suggests revisiting CT at 12-24 months or sooner if symptoms or PFT progress [7]. Spirometry, lung volumes, and diffusion capacity (DLCO) are suggested PFTs. Honeycombing or traction bronchiectasis should be directed to a pulmonologist regardless of diffusion ability, according to experts. With confirmed ILA but no PFT abnormalities, over 90% of expert panel members advocated follow-up, specialist referral, or periodic repeat testing [9,10]. Testing for the MUC5B promoter polymorphism (rs35705950) is relevant given its strong association with ILA progression and IPF [24]. While not universally recommended for all ILAs, it can help identify high-risk individuals.

There are some conflicts in follow-up protocols. While expert consensus exists for monitoring high-risk ILAs, there are still controversies and a lack of specific, agreed-upon protocols for low-risk groups [12]. For asymptomatic patients with non-dependent subpleural reticulation, honeycombing/traction bronchiectasis, or centrilobular ground-glass nodules/patchy ground-glass opacity, consensus was not met regarding the type of follow-up testing (PFTs alone vs. PFTs + HRCT). The "wait-and-see" approach currently recommended for some low-risk ILA is based on weak evidence [9,10].

Clinical Risk Scoring Tool for Follow-up Prioritization

**Figure 3:**
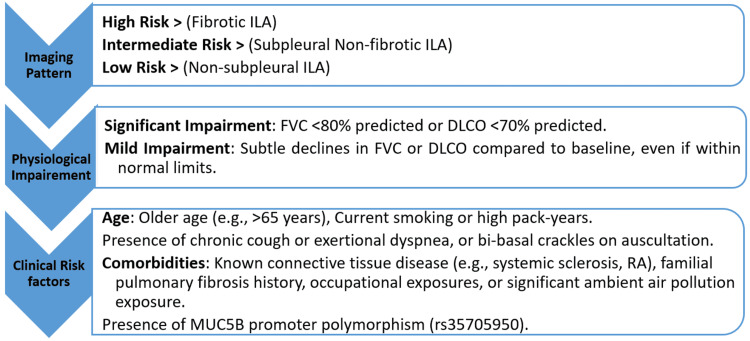
Algorithm for follow-up prioritization. FVC: forced vital capacity; ILA: interstitial lung abnormalities; DLCO: diffusing capacity of the lungs for carbon monoxide; RA: rheumatoid arthritis This figure is created by the authors of this study.

Integration of the following factors, illustrated in Figure 3, can be beneficial for a clinical risk scoring tool for follow-up prioritization based on literature evidence [9].

Proposed Prioritization Algorithm

**Figure 4:**
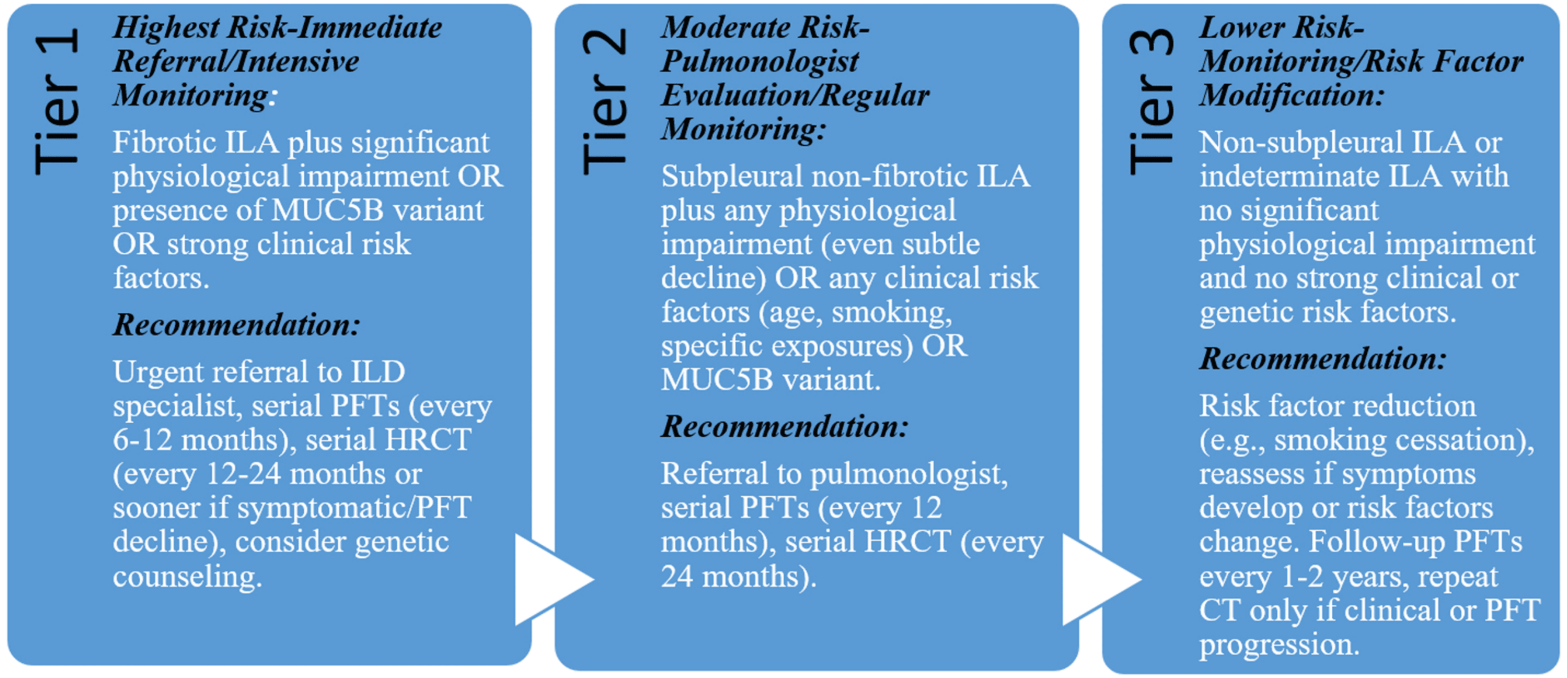
Proposed prioritization algorithm based on literature synthesis. ILA: interstitial lung abnormalities; ILD: interstitial lung disease; HRCT: high-resolution computed tomography scan; PFT: pulmonary function test This figure is created by the authors of this study.

The algorithm presented in Figure 4 enables stratification for individualized follow-up, optimizes resource allocation, and aims for early intervention [9].

Translational and research implications

The growing insights into ILAs open several translational and research avenues, including the question of whether early intervention in subclinical disease is feasible. The beneficial effects of antifibrotic agents in IPF, even in patients with preserved lung function, have prompted interest in early intervention for subclinical IPF. However, robust clinical trials specifically targeting ILAs with antifibrotic therapy are currently lacking. The feasibility of early intervention is a major research question, aiming to potentially reduce disease progression and mortality in high-risk ILA subsets [10,28].

Although promising literature indicates that antifibrotic therapy can reduce the rate of lung-function decline in patients with IPF and other progressive fibrosing ILD, including those with preserved lung function, whether patients with early-stage ILA would also benefit is still under investigation. This is a key area for future clinical trials [9,10,12]. However, artificial intelligence (AI) and machine learning (ML) methods are being developed for automatic identification and classification of ILD patterns and ILAs on CT images. These tools can overcome limitations of subjective visual assessment, such as inter-reader variability. Deep learning-based texture analysis has shown high sensitivity (100%) and specificity (99%) for detecting ILA using a 1.8% lung area cutoff value, which is significantly lower than the conventional 5% threshold, enabling detection of even more subtle changes [18]. This can improve objective recognition of ILAs in screening CTs. AI-based quantification of pulmonary fibrosis from CT images has been shown to be an independent predictor of disease-free survival in lung cancer patients [12].

Future directions

Large-scale prospective trials are urgently needed to clarify the natural history of ILA progression, especially for subpleural non-fibrotic types, and to validate optimal follow-up strategies. Biomarker identification is a reliable blood-based biomarker, which is a high priority [9,12]. Surfactant protein D (SPD) has shown diagnostic potential for HRCT abnormalities in ACPA-positive subjects, and SP-B has been significantly associated with ILA and its progression. Matrix metalloproteinases (MMPs), resistin, IL-6, CRP, and growth differentiation factor 15 (GF-15) are also being investigated as ILA biomarkers. Further research is needed to identify biomarkers that can accurately predict progression to IPF from the broader ILA category [12,18,40].

Highlighting AI-based predictive models and deep-learning CT analysis as next-gen tools

AI and deep learning CT analyses are poised to revolutionize the field of ILA management. They offer the capability to automate detection and quantification. Deep learning models can objectively identify and quantify interstitial abnormalities, potentially with higher sensitivity than visual inspection, and reduce inter-observer variability. For example, using deep learning, a system identified and classified ILA patterns with 91.4% sensitivity and 98.18% specificity. To predict progression and risk, AI-based predictive models can analyze CT features and potentially integrate other clinical and genetic data to forecast ILA progression and mortality risk more accurately [12,18]. This would allow for better risk stratification and follow-up prioritization. By identifying subtle patterns and complex relationships, these tools could contribute to a precision medicine approach, guiding early therapeutic decisions based on an individual's unique disease signature. The Framingham Heart Study CT cohorts, for instance, are being utilized to establish machine learning models and artificial intelligence radiology. These advanced computational methods promise to bridge the current knowledge gaps, leading to earlier diagnosis and potentially more effective interventions for progressive fibrotic lung diseases [19,20].

## Conclusions

In asymptomatic adults, subclinical interstitial lung abnormalities (ILAs) were considered incidental findings. It is now increasingly being recognized that these are at least predisposing factors to early-stage interstitial lung disease, yet their clinical implications are unclear. The incidence rises with age and exposure to smoking; however, much of that evidence lacks longitudinal follow-up studies that are detailed enough to distinguish mild ILAs with benign courses and progressive disease-producing ILAs. The asymptomatic ILA management is not standardized because there is a lack of universally accepted diagnostic criteria, risk stratification assessment tools, and clearly formulated instructions for surveillance. To transform ILAs as incident discoveries into some clinically actionable data, there is a need to widen the views by prospective studies, the generation of biomarkers, and investigation in long-duration follow-ups.
